# PLK1 inhibition enhances temozolomide efficacy in IDH1 mutant gliomas

**DOI:** 10.18632/oncotarget.15015

**Published:** 2017-02-02

**Authors:** Robert F. Koncar, Zhengtao Chu, Lindsey E. Romick-Rosendale, Susanne I. Wells, Timothy A. Chan, Xiaoyang Qi, El Mustapha Bahassi

**Affiliations:** ^1^ Department of Internal Medicine, Division of Hematology/Oncology, University of Cincinnati, Cincinnati, OH, USA; ^2^ Division of Oncology, Cincinnati Children's Hospital Medical Center, Cincinnati, OH, USA; ^3^ Human Oncology and Pathogenesis Program, Memorial Sloan-Kettering Cancer Center, New York, NY, USA

**Keywords:** glioma, IDH1, temozolomide, PLK1, checkpoint adaptation

## Abstract

Despite multimodal therapy with radiation and the DNA alkylating agent temozolomide (TMZ), malignant gliomas remain incurable. Up to 90% of grades II-III gliomas contain a single mutant isocitrate dehydrogenase 1 (*IDH1*) allele. *IDH1* mutant-mediated transformation is associated with TMZ resistance; however, there is no clinically available means of sensitizing *IDH1* mutant tumors to TMZ. In this study we sought to identify a targetable mechanism of TMZ resistance in *IDH1* mutant tumors to enhance TMZ efficacy. *IDH1* mutant astrocytes rapidly bypassed the G2 checkpoint with unrepaired DNA damage following TMZ treatment. Checkpoint adaptation was accompanied by PLK1 activation and *IDH1* mutant astrocytes were more sensitive to treatment with BI2536 and TMZ in combination (<20% clonogenic survival) than either TMZ (~60%) or BI2536 (~75%) as single agents. *In vivo*, TMZ or BI2536 alone had little effect on tumor size. Combination treatment caused marked tumor shrinkage in all mice and complete tumor regression in 5 of 8 mice. Mutant *IDH1* promotes checkpoint adaptation which can be exploited therapeutically with the combination of TMZ and a PLK1 inhibitor, indicating PLK1 inhibitors may be clinically valuable in the treatment of *IDH1* mutant gliomas.

## INTRODUCTION

Malignant gliomas are currently associated with a dismal prognosis and recurrence remains nearly inevitable despite a multimodal treatment strategy [1, 2]. Gliomas are histologically graded as I-IV and 70-90% of grades II-III gliomas and secondary grade IV glioblastomas contain a mutation in one *Isocitrate dehydrogenase 1* (*IDH1*) allele, with R132H being the most common [3–5]. IDH1 is found in the cytoplasm and peroxisomes where it converts isocitrate to alpha ketoglutarate (αKG). However, the mutant enzyme converts αKG into oncometabolite D-2-hydroxyglutarate (D2HG), which is structurally similar to αKG and a competitive inhibitor of αKG-dependent dioxygenases [6–8].

Treatment for gliomas typically consists of surgical resection, radiation therapy, and chemotherapy with the DNA alkylating agent, temozolomide (TMZ) [9]. The cytotoxic effect of TMZ is mediated primarily through generating O-6-methylguanine (O6meG) lesions [10]. If the methyl group is not removed by O-6-methylguanine-DNA methyltransferase (MGMT), an enzyme associated with TMZ resistance, O6meG mispairs with thymine during DNA replication, leading to futile rounds of mismatch repair and persistent G2 checkpoint arrest followed by apoptosis or senescence [11]. MGMT promoter methylation and consequently low MGMT expression is typical in, but not unique to *IDH1* mutant gliomas [12], which generally respond better to TMZ than their *IDH1* wild type (WT) counterparts [13, 14]. However, MGMT expression is not the sole determinant of TMZ sensitivity [15–18] and *IDH1* mutant and wild-type gliomas have different molecular ontogenies, making comparisons between *IDH1* mutant and wild type gliomas uninformative as to which tumor characteristics can be attributed directly to *IDH1* mutation. Grade II-III gliomas lacking the *IDH1* mutation are genetically distinct from *IDH1* mutant gliomas and are more similar to primary grade IV glioblastomas. While genetic alterations such as *EGFR* amplification and *CDKN2A* deletion are common in *IDH1* WT gliomas, they rarely occur in gliomas with mutant *IDH1* [19]. Despite being considered chemoresponsive IDH1 mutant gliomas commonly recur even after surgical resection and treatment with radiation and temozolomide, highlighting the need for new treatment options [20–22].

Recent evidence suggests that *IDH1* mutant-mediated transformation promotes TMZ resistance and rapid G2 checkpoint exit due to increased homologous recombination capability [23]. How IDH1 affects DNA repair and checkpoint signaling however, is unknown. The DNA damage checkpoint is a critical process that coordinates cell cycle progression with DNA damage repair. Thus, understanding how *IDH1* mutation affects checkpoint signaling may reveal ways to further sensitize IDH1 mutant tumor cells to TMZ.

Polo-like kinase 1 (PLK1) is a key regulator of mitotic progression following DNA damage-induced G2 checkpoint activation. It is involved in checkpoint recovery, which requires repair of damaged DNA, and checkpoint adaptation, in which cell division occurs with unrepaired DNA damage [24]. PLK1 is commonly overexpressed or over-activated in cancer, and is the target of several promising drugs in late stage clinical trials [25].

In this study, we sought to elucidate the mechanism of TMZ resistance and to identify potential targets to enhance TMZ efficacy in IDH1 mutant tumors. To this end, we used immortalized astrocytes to ask whether mutant IDH1 promotes TMZ resistance as a consequence of D2HG production and whether checkpoint adaptation, mediated through PLK1 activation rather than swift DNA damage repair accounts for the early progression out of G2 arrest. We show that IDH1 mutant cells and tumors can be greatly sensitized to TMZ by inhibiting PLK1 *in vitro*, as well as in a xenograft mouse model.

## RESULTS

### IDH1 mutant-associated D2HG promotes TMZ resistance

**Figure 1:**
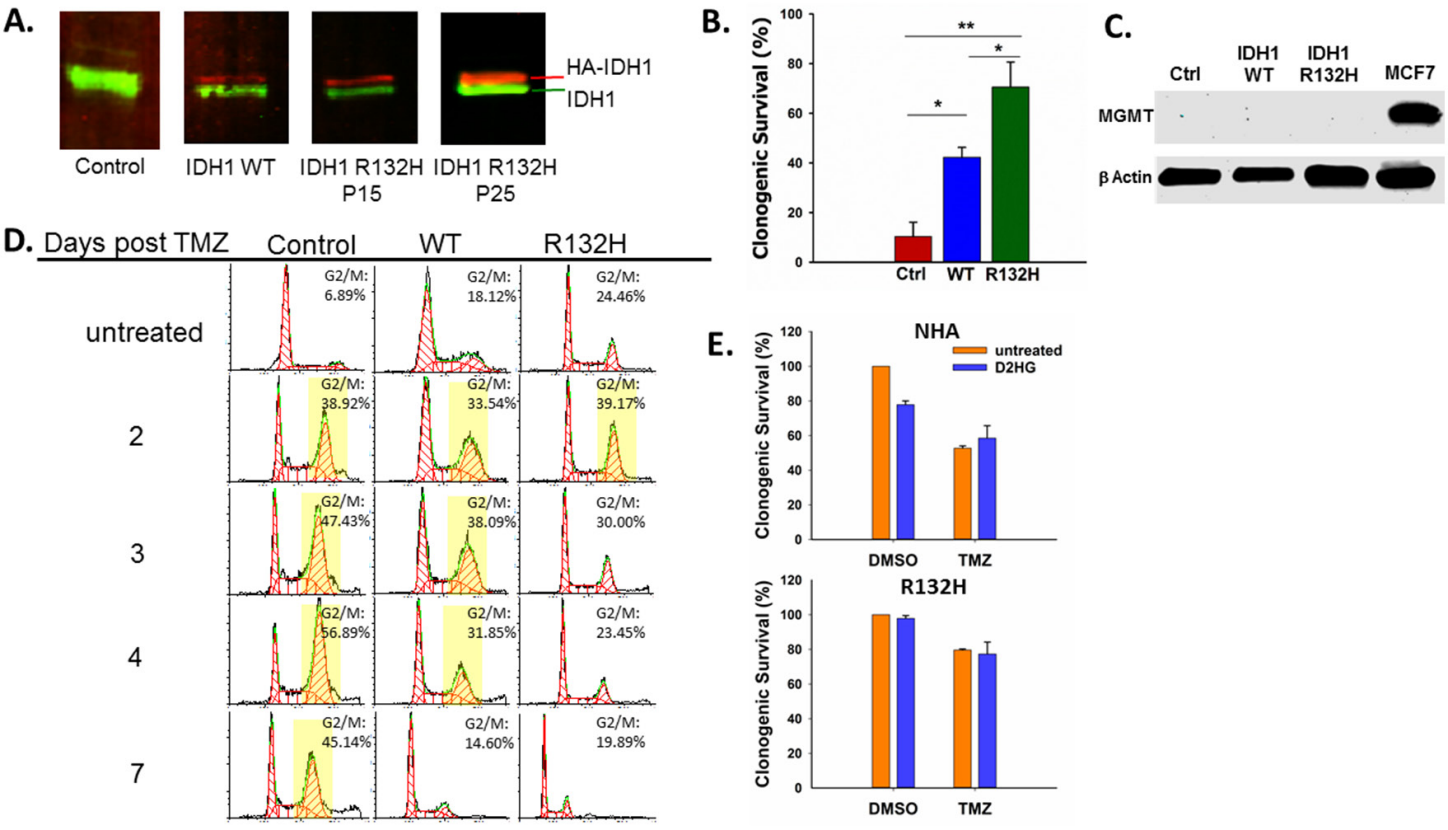
IDH1 mutation promotes resistance to TMZ by D2HG production **A**. Western blot confirming expression of exogenous HA-IDH1 (red) and endogenous IDH1 (green). **B**. Clonogenic survival of empty vector control, IDH1 WT, and IDH1 mutant astrocytes after treatment with 100μM TMZ. **C**. MGMT expression was not detectable by Western blot in astrocytes regardless of IDH1 status. MCF7 cells were used as a positive control. **D**. Impact of mutant IDH1 on cell cycle profiles in response to TMZ treatment. Yellow boxes indicate>30% of cells in G2/M. **E**. Clonogenic survival of parental astrocytes (top) and IDH1 mutant astrocytes (bottom) cultured with or without 5mM D2HG and treated with TMZ. There was a statistically significant interaction between D2HG and TMZ treatments in the NHA (P=0.02) but not in IDH1 mutant astrocytes. Error bars represent SEM. P<0.05 (*); P<0.01 (**).

To study the effects of mutant *IDH1*, we used normal human astrocytes (NHA) which have been immortalized and described elsewhere [26]. When transformed by expression of an exogenous mutant *IDH1* gene, the NHA epigenetically resemble IDH1 mutant gliomas [27]. A hemagglutinin (HA) tagged WT or R132H mutant IDH1 gene was introduced into the NHA by retroviral transduction and gene expression was confirmed by Western blot (Figure 1A). WT and IDH1 R13H clones showing comparable levels of exogenous wild type and mutant IDH1 proteins were selected. The WT and mutant cell lines were additionally confirmed by Sanger sequencing (Supplementary Figure 1A). NMR spectroscopy revealed increased 2HG concentrations in the IDH1 mutant cells (Supplementary Figure 1B).

After confirming the presence of the IDH1 mutation and 2HG production by the astrocytes we used them to test the effect of IDH1 mutation on TMZ sensitivity by clonogenic survival. After treatment with TMZ (100μM), mutant IDH1 NHA were significantly less sensitive to TMZ while WT NHA displayed an intermediate phenotype between the control and IDH1 mutant cells (Figure 1B), which is consistent with published data [23]. Differences in TMZ sensitivity were not due to differential MGMT expression as all three cell lines were MGMT deficient (Figure 1C).

The response of an MGMT deficient cell line such as NHA to TMZ is prolonged G2 arrest [11, 23]. This characteristic arrest was seen in the control NHA which displayed sustained G2 arrest up to 7 days after TMZ treatment (Figure 1D). However, IDH1 mutant NHA displayed a much shorter G2 arrest and by day 3 post TMZ treatment, the percentage of cells in G2 was similar to that of untreated cells (Figure 1D). IDH1 WT NHA again displayed an intermediate phenotype between the mutant and control NHA with sustained G2 arrest through Day 4 post TMZ.

Production of D2HG is considered to be the primary means by which mutant IDH1 promotes oncogenesis [7, 8, 28]. D2HG is required for maintenance of oncogenic properties of IDH1 mutant cells and has been shown to inhibit the activity of αKG-dependent dioxygenases which may play a role in cellular transformation [6, 7, 28]. To determine if TMZ resistance conferred by IDH1 mutation is mediated by D2HG production, we evaluated TMZ sensitivity in IDH1 mutant and parental NHA treated with D2HG, which has been shown to rapidly enter cells [29], at a concentration (5mM) within the range of what has been reported in IDH1 mutant gliomas [6, 30]. In parental NHA, D2HG alone resulted in a mild but significant decrease in survival which is consistent with reported effects of D2HG treatment on glioma cell lines [29]. However, D2HG treatment resulted in increased survival of parental NHA treated with TMZ (Figure 1E), and there was a significant interaction between TMZ treatment and D2HG treatment (P=0.02), indicating D2HG production acutely promotes TMZ resistance. D2HG treatment had no effect on the IDH1 mutant astrocytes.

### IDH1 mutation promotes premature G2 checkpoint exit following TMZ treatment

**Figure 2:**
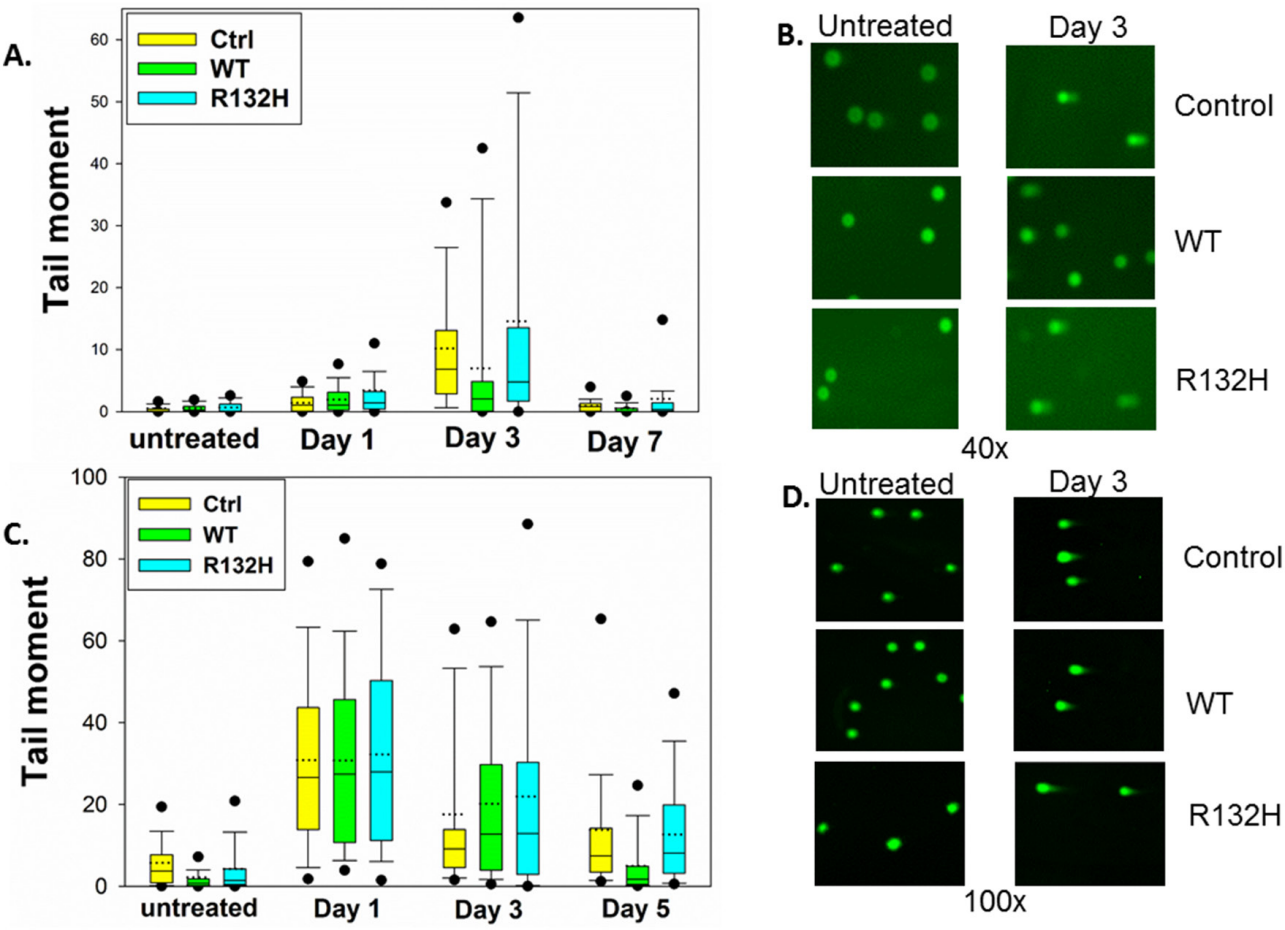
IDH1 mutation does not affect levels of DNA damage following TMZ treatment **A**. Alkaline comet assay reveals no significant difference in total DNA damage between control, IDH1 WT, or IDH1 mutant astrocytes at day 1, 3, or 7 post TMZ treatment. **B**. Representative images of alkaline comet assay three days after TMZ treatment. **C**. No significant difference in double-strand DNA breaks was detected by neutral comet assay. **D**. Representative images of neutral comet assay three days post TMZ. Box plots represent median, first, and third quartiles. Bars show 10^th^ and 90^th^ percentiles. Circles show 5^th^ and 95^th^ percentiles.

DNA damage-induced G2 checkpoint arrest is followed by checkpoint recovery, checkpoint adaptation, or apoptosis. While checkpoint recovery allows cell cycle progression after repair of damaged DNA, checkpoint adaptation allows mitotic progression despite unrepaired DNA damage [24]. To determine whether the shortened TMZ-induced G2 arrest in *IDH1* mutant cells is due to efficient repair of damaged DNA or premature checkpoint override with residual unrepaired DNA damage, we measured total DNA damage by alkaline comet assay. IDH1 mutant astrocytes did not show statistically different levels of DNA damage than control and IDH1 WT astrocytes at days 1, 3, and 7 post TMZ (Figure 2A, 2B). We next specifically examined the more lethal double-strand breaks by neutral comet assay, and again found no statistically significant differences between the three cell types (Figure 2C, 2D), even at three and five days after TMZ treatment, when IDH1 mutant cells have already exited the G2 checkpoint (Figure 1D). These data indicate that IDH1 mutant astrocytes prematurely exit the G2 checkpoint with unrepaired DNA damage and that progression into mitosis is facilitated by checkpoint adaptation rather than checkpoint recovery.

### Inhibition of polo-like kinase 1 sensitizes IDH1 mutant astrocytes to TMZ

**Figure 3:**
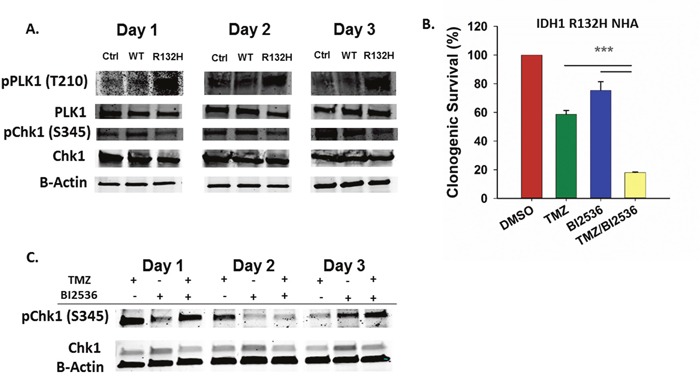
Inhibition of PLK1 sensitizes IDH1 mutant cells to TMZ **A**. Western blot using phospho-specific antibodies shows increased PLK1 activation and decreased CHK1 activation in mutant IDH1 astrocytes after TMZ treatment compared to control and WT astrocytes. **B**. Clonogenic survival of IDH1 mutant astrocytes following treatment with TMZ and a PLK1 inhibitor, BI2536. **C**. Western blot for total and activate CHK1 in IDH1 mutant astrocytes treated with TMZ and BI2536. Error bars represent SEM. n=3. P<0.001 (***).

PLK1 regulates G2 checkpoint adaptation and progression into mitosis following DNA damage and acts antagonistically to the CHK1 signaling pathway which is essential for maintenance of G2 checkpoint arrest [31–35]. Aberrant PLK1 activation can facilitate G2 checkpoint bypass and repress apoptotic signaling pathways, allowing cells to divide and survive despite failing to complete repair of damaged DNA [24, 36, 37]. One mechanism by which PLK1 inactivates the G2 DNA damage checkpoint is through phosphorylation of the CHK1 regulatory protein Claspin, which targets it for degradation and leads to the inactivation of the ATR/CHK1 signaling pathway [34]. Examination of PLK1 and CHK1 phosphorylation in TMZ-treated cells revealed elevated PLK1 activation and diminished CHK1 activation in the *IDH1* mutant astrocytes (Figure 3A).

Several PLK1 inhibitors are currently in clinical trials as cancer therapeutics [25]. To determine if inhibition of PLK1 sensitizes *IDH1* mutant astrocytes to TMZ, we treated cells with BI2536, a potent and selective PLK1 inhibitor. Co-treatment of IDH1 mutant cells with TMZ and BI2536 resulted in more than a threefold decrease in clonogenic survival rate when compared to TMZ or BI2536 treatment alone (P<0.001) (Figure 3B). Compared to TMZ alone, treatment with BI2536 and TMZ also increased CHK1 activation at day 3 post TMZ (Figure 3C). Combination treatment of IDH1 WT cells resulted in a less dramatic, though significant reduction in clonogenic survival when compared to treatments of either TMZ or BI2536 alone (Supplementary Figure 2).

### A new *in vivo* model of IDH1 mutant glioma

Since inhibition of PLK1 sensitized IDH1 mutant NHA to TMZ *in vitro*, we sought to determine whether a combination of TMZ and BI2536 is more effective than either single drug *in vivo*. However, cells from the majority of IDH1 mutant gliomas do not grow *in vitro* and tumors passaged directly in mice do not consistently retain their original characteristics [38–40]. We assessed the tumorigenic potential of the IDH1 mutant astrocytes in subcutaneous and orthotopic xenograft mouse models. Astrocytes expressing mutant *IDH1* for either 15, 25, or 50 passages (P15, P25, P50, respectively) were injected subcutaneously in mice. The astrocytes were all of approximately the same total passage number, and differed only in the number of passages with the *IDH1* mutant gene. All mice injected with the *IDH1* mutant cells formed tumors. However, the time to tumor formation depended on the number of passages for which the cells expressed mutant *IDH1* (Supplementary Figure 3A). The P50 cells formed tumors by 11 weeks, while P25 cells averaged almost 13 weeks, and P15 cells averaged more than 18 weeks to form tumors (Supplementary Figure 3A, 3B). Targeted sequencing revealed retention of wild-type and mutant *IDH1* alleles in the tumors (Supplementary Figure 3C).

P50 cells were also transduced with a constitutively active luciferase reporter and tested for orthotopic tumorigenicity in the mouse brain. The first tumor was detectable by six weeks after injection and was fatal by week 9 (Supplementary Figure 3D). Of 12 mice injected, only three formed tumors that were clearly detectable by luminescence. We therefore chose to test the therapeutic treatments in the subcutaneous model.

### BI2536 enhances TMZ anti-tumor efficacy *in vivo*

**Figure 4:**
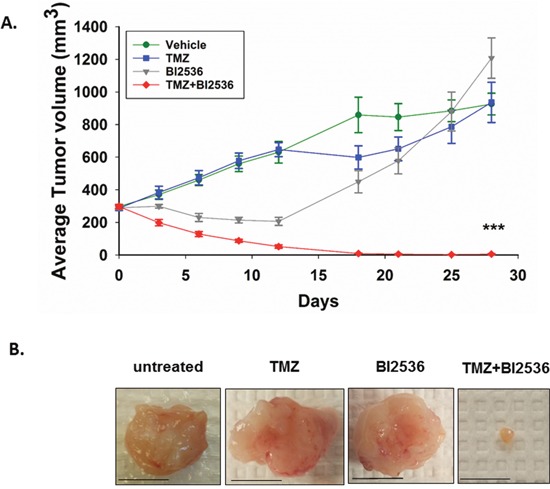
Combination of TMZ and BI2536 leads to a marked tumor regression in IDH1 mutant tumors **A**. Average subcutaneous tumor volumes of vehicle, TMZ, BI2536, and TMZ+BI2536-treated mice over 28 days. **B**. Representative images of excised subcutaneous tumors 31 days after treatment began. Scale bars=1cm. Error bars represent SEM. P<0.001 (***). n≥5 mice per treatment group.

After establishing that IDH1 mutant astrocytes form tumors efficiently as subcutaneous xenografts, we tested TMZ and BI2536 as a combination treatment for subcutaneous, IDH1 mutant tumors in NOD-*scid* IL2Rgamma^null^ mice. Mice were injected twice, three days apart, with 80mg/kg of TMZ and 40mg/kg BI2536 either alone or in combination, or with vehicle. Tumor volumes were tracked for 28 days. The combination therapy had significantly greater anti-tumor efficacy than TMZ or BI2536 as monotherapies (day 28 P<0.001). Treatment with the combination of both drugs produced a remarkable reduction in tumor size. At day 28, tumors in TMZ or BI2536 treated mice were similar in size to those of the vehicle treated mice (Figure 4A, 4B). Notably, five of eight mice treated with both drugs exhibited complete tumor regression. In the three cases where tumor regression was not complete, the tumor shrank markedly (Figure 4B), with no tumor exceeding 20mm^3^ at day 28. In contrast, each of the other three treatment groups had an average tumor volume exceeding 900mm^3^ at day 28 (Figure 4A). Mice receiving the combination treatment showed no obvious signs of toxicity and lost no more than 10% body weight at any point in time (Supplementary Figure 4).

### PLK1 inhibitor plus TMZ combination is effective in IDH1 mutant patient-derived cells

After testing the combination therapy in IDH1 mutant astrocytes, we were able to obtain GBM164 cells which are patient derived cells that are maintained as xenografts but can be cultured for several passages *in vitro* [41]. The cells were genotyped and confirmed to be *IDH1* heterozygous mutant (Supplementary Figure 5A). To confirm that the effect of combination treatment with BI2536 and TMZ is not unique to our astrocyte model, a cell viability assay was performed on GBM164 cells. Combination treatment resulted in over a seven fold decrease in cell viability compared to TMZ (P≤0.001) or BI2536 (P=0.002) alone (Supplementary Figure 5B).

## DISCUSSION

In gliomas, when the *IDH1* mutation is present, it is typically found throughout the entirety of otherwise heterogeneous tumors, which makes targeting vulnerabilities conferred by mutant *IDH1* very appealing [5]. In the current work, we report that the *IDH1* mutation promotes TMZ resistance through G2 checkpoint adaptation facilitated by PLK1 activation. Additionally, treatment with a PLK1 inhibitor dramatically improves TMZ efficacy while establishing the use of *IDH1* mutant astrocytes in a xenograft mouse model.

Our data indicate that in the context of an *IDH1* mutation, PLK1 activation promotes bypass of the TMZ-induced DNA damage checkpoint, limiting TMZ effectiveness. PLK1 can inactivate the DNA damage checkpoint by inactivating or facilitating degradation of target proteins such as Claspin, a mediator of CHK1 activation and G2 checkpoint maintenance [34, 35]. CHK1 and PLK1 act antagonistically as PLK1 can be inactivated in a CHK1 activity-dependent manner [31, 32]. Consistent with this model, our data confirm that CHK1 is inactivated and PLK1 is activated in *IDH1* mutant cells following TMZ exposure.

Interestingly, TMZ resistance has been linked to IDH1 mutant-mediated transformation, rather than the immediate activity of the mutant enzyme [23]. Clearly, the long-term and immediate effects of IDH1 mutation are not mutually exclusive and while the indirect effects of an IDH1 mutation likely play a role, we show that D2HG also acutely promotes TMZ resistance.

While the mechanism of PLK1 activation in *IDH1* mutant tumors is still under investigation, we postulate that the immediate effect may be in part through the inhibition of αKG-dependent enzymes. D2HG produced by mutant IDH1 inhibits members of the TET and JmjC families of enzymes, which are regulators of DNA and histone methylation, respectively. TET inhibition is associated with DNA hypermethylation resulting in the CpG island methylation phenotype and altered gene expression profile. However, no significant changes in expression or DNA methylation occur at *PLK1* or *CHK1* loci in the *IDH1* mutant astrocytes [27]. Additionally, genes for upstream regulators of PLK1 and CHK1 such as Aurora A, Bora, and ATR also remain unaffected. A single CpG island locus in the *ATRIP gene* was reported to be slightly hypomethylated, though gene expression is not significantly affected [27].

Alternatively, it is possible that D2HG promotes PLK1 activation by inhibiting Egln3 activity. Hydroxylation of Telo2 by the αKG-dependent dioxygenase Egln3 is required for activation of the ATR/CHK1 checkpoint pathway which in turn leads to PLK1 inactivation [42, 43]. However, D2HG competes with αKG as an Egln3 substrate, possibly leading to inhibition of Egln3 activity and ultimately to CHK1 inactivation and PLK1 activation. Inhibition of Egln3 and the corresponding decrease in Telo2 hydroxylation has also been shown to decrease apoptosis following DNA damage [42].

αKG-dependent dioxygenases may also account for the mild temozolomide (TMZ) resistance in the IDH1 WT NHA. While IDH1 mutant NHA display hypermethylation as a result of TET inhibition, Turcan et al. reported genomic hypomethylation in IDH1 WT NHA suggesting that αKG production may enhance αKG-dependent dioxygenase activity [27]. It is possible that IDH1 WT NHA repair TMZ-induced DNA damage more efficiently by activating EGLN3 or members of the AlkBH family of αKG-dependent DNA repair enzymes. Improved DNA repair capacity in the IDH1 WT NHA would account for the shortened G2 arrest (Figure 1D) and fewer double strand breaks at day 5 post TMZ (Figure 2C).

Increased homologous recombination has also been proposed as the mechanism of TMZ resistance in mutant IDH1-transformed astrocytes [23]. However, we demonstrate that *IDH1* mutant cells have no less DNA damage than IDH1 WT and control cells, indicating checkpoint adaptation rather than recovery is responsible for early G2 checkpoint exit. While PLK1 has been shown to phosphorylate BRCA1 and RAD51 to promote homologous recombination, [44, 45] it is possible that the *IDH1* mutation and PLK1 activation not only promote homologous recombination, or at least activation of the homologous recombination machinery, but also premature mitotic progression prior to complete repair of damaged DNA.

To date, all work concerning the use of temozolomide (TMZ) in combination with a PLK1 inhibitor has been in the context of primary grade IV glioblastoma, which rarely carries an *IDH1* mutation [46–48]. Compared to their wild type counterparts, IDH1 mutant gliomas have a favorable clinical response to TMZ and IDH1 has become an important prognostic factor [49]. Importantly, IDH1 wild-type and mutant gliomas are molecularly very different and comparative clinical outcome does not indicate how mutant IDH1 affects a tumor's response to treatment. Additionally, our work demonstrates the context-dependent effects of mutant *IDH1* which has previously been reported to either increase or have no effect on TMZ sensitivity when expressed in U87 and U373 cell lines [50, 51]. In contrast, we and others have shown that the expression of mutant IDH1 in untransformed cells promotes cellular transformation and temozolomide (TMZ) resistance [23].

The sensitivity of *IDH1* mutant tumors to a PLK1 inhibitor plus TMZ combination may have implications beyond glioma treatment. *IDH* mutation is found frequently in acute myeloid leukemia, cholangiocarcinoma, osteosarcoma, and in central and periosteal chondrosarcomas which are nonresponsive to current chemotherapy regimens [52]. Interestingly, a variety of PLK1 inhibitors are in clinical and preclinical development with two of them in late clinical trials [25]. Our findings establish PLK1 as a very promising target in IDH1 mutant tumors and warrant consideration of clinical trials for TMZ and PLK1 inhibitor combinations.

## MATERIALS AND METHODS

### Cell lines

Immortalized human astrocytes have been described elsewhere [26]. HA-tagged wild-type and R132H mutant IDH1 were cloned into pBABE-neo retroviral vector. Astrocytes were transduced with retrovirus and underwent G418 selection. Clones were screened for expression of HA-IDH1. IDH1 mutant astrocytes used at passage50 were generated as previously described [27].

GBM164, MCF7, and all NHA cell lines were grown as adherent cells in DMEM, 10% FBS.

### Western blotting

Western blotting was performed using Bio-Rad mini-protean®TGX™ gels and PVDF membranes. Primary antibodies used: anti-IDH1 (N-20;#sc-49996, Santa Cruz), anti-pPLK1 T210 (D5H7;#9062, Cell Signaling), anti-PLK1 (208G4, #4513, Cell Signaling), anti-pCHK1 S345 (133D3;#2348, Cell Signaling), anti-CHK1 (2G1D5, #2360, Cell Signaling), anti-β Actin (8H10D10, #3700, Cell Signaling), anti-HA (6E2, #2367, Cell Signaling), and anti-MGMT (OAAF03046, Aviva Systems Biology). Imaging was performed on a LI-COR Odyssey imager.

### Nuclear magnetic resonance spectroscopy

Cell collections and extractions were performed as previously described [53]. Briefly, the hydrophilic cell extract samples were dried in a SpeedVac centrifuge then resuspended in NMR buffer. One-dimensional ^1^H NMR spectra were recorded using Carr-Purcell-Meiboon-Gill pulse sequence with presaturation of the water peak on a 600 MHz INOVA spectrometer. Experiments were run with 4 dummy scans and 128 acquisition scans (acquisition time: 2.09s, relaxation delay: 4.0s, mixing time: 60ms). Spectral width was 26ppm, and 64K real data points were collected. Pure D2HG was used to confirm the metabolite of interest. All NMR data were processed using TopSpin3.1 (Bruker Analytik, Rheinstetten, Germany). All FIDs were subjected to exponential line-broadening of 0.3 Hz. Upon Fourier transformation, each spectrum was manually phased, baseline corrected, and referenced to the internal standard TMSP at 0.0ppm.

### Clonogenic survival assay

Cells (1 × 10^3^) were plated on 10cm plates. When adhered, cells were treated with DMSO or 2nM BI2536 (Selleck) for 42hrs, 100μM temozolomide (Cayman) for 18hrs, or 100μM TMZ+2nM BI2536 for 18hrs followed by an additional 24hrs with 2nM BI2536 so that BI2536 was present during the second cell cycle following TMZ exposure. The day after TMZ was washed out was designated day 1 post TMZ. When D2HG treatment was included, cells were incubated with complete media plus 5mM D2HG from 8hrs prior to TMZ treatment until cells were fixed and stained. Cells were then grown in DMEM, 10% FBS until colonies clearly formed in DMSO-treated plates. The plates were washed, fixed with 4% paraformaldehyde, and stained with 0.1% crystal violet.

### Cell viability assay

In a 96 well plate, 5 × 10^3^ GBM164 cells were plated per well. Two days later cells were treated with 200μM TMZ and 4nM BI2536 and incubated for three days, after which treatment was washed out and cell viability was measured using cell counting kit 8 (Dojindo Laboratories, Rockville, MD) according to the manufacturer's protocol.

### Propidium iodide staining/cell cycle analysis

After 18hrs TMZ treatment, cells were washed and incubated with complete media until collection. Cells were trypsinized, washed, fixed with cold 70% ethanol, and propidium iodide stained. Flow cytometry was performed on a BD-LSR-Fortessa flow cytometer. Analysis was performed on FCS Express4 with≥10^4^ events per time point.

### Comet assay

The Trevigen Comet assay kit (4250-050-K) was used according to manufacturer's protocol. All samples for a given time point were run together in duplicate. DNA was stained with SYBR Gold (LifeTechnologies), viewed with an Olympus-BX51 microscope, imaged with a SPOT-RT-KE camera (Diagnostic Instruments), and ≥40 comets were scored for each sample using OpenComet [54].

### Testing drug efficacy *in vivo*

Five-week-old female NOD-*scid* IL2Rgamma^null^ mice were injected with 2.5×10^6^ cells subcutaneously in the right flanks. Treatments were administered when tumors reached 300mm^3^, and again three days later. Size was determined with calipers and volume was calculated by: V=LxWxH/2. Temozolomide (80mg/kg) was injected intraperitoneally in 10% DMSO. BI2536 (40mg/kg) was dissolved in 0.02N HCl and administered by tail vein injection.

### Intracranial xenograft

Intracranial xenografts were established as previously described [55]. Briefly, five-week-old female NOD-*scid* IL2Rgamma^null^ mice were anesthetized, fixed in a stereotactic apparatus, and 10^5^ cells constitutively expressing luciferase were injected with a Hamilton syringe 2mm lateral to the bregma point at a depth of 3mm. Mice were imaged with a Bruker *In-Vivo* MS FX PRO. All mouse work was in accordance with a protocol approved by the University of Cincinnati Institutional Animal Care and Use Committee.

### Statistical analysis

Significance was set to P≤0.05 for all experiments. One-way ANOVA was used to compare between groups. To test for treatment interactions, two way ANOVA was used. The Holm-Sidak method was used for post-hoc testing. All statistical analyses were performed with SigmaPlotV13.

## SUPPLEMENTARY FIGURES


