# Supplementary material for: PLK1 inhibition enhances temozolomide efficacy in IDH1 mutant gliomas
Source: Oncotarget. 2017 Feb 2;8(9):15827–37. doi: 10.18632/oncotarget.15015 (PMC5362526; doi:10.18632/oncotarget.15015)
Supplement: Supplementary file 1 [file oncotarget-08-15827-s001.pdf]

PLK1 inhibition enhances temozolomide efficacy in IDH1 mutant gliomas

SUPPLEMENTARY FIGURES

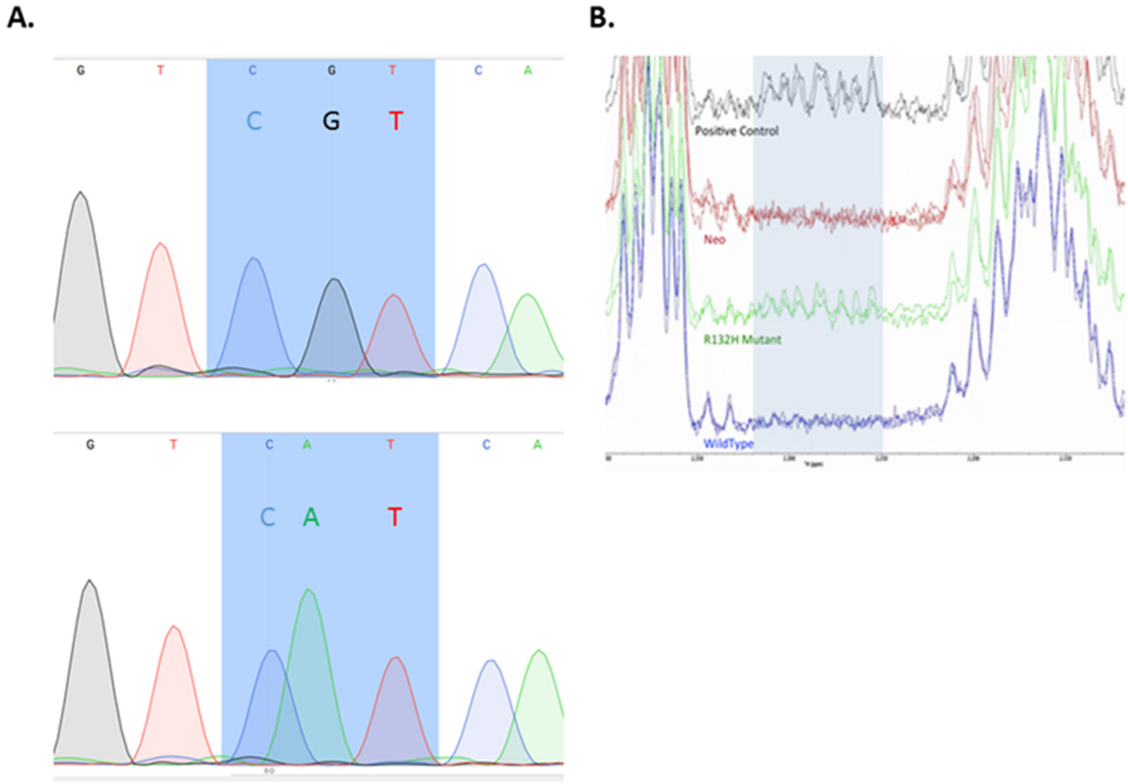

Supplementary Figure 1: Confirmation of IDH1 WT and R132H mutant cell lines. A. Targeted sequencing of IDH1 cDNA sequence from gDNA of IDH1 WT (top) and R132H mutant transduced astrocytes (bottom). Codon 132 is highlighted in blue. **B.** NMR spectroscopy was unable to detect 2HG in control (red) and IDH1 WT astrocytes (blue). 2HG is clearly detected (seven peaks highlighted in blue) in R132H mutant astrocytes (green) and positive control (black).

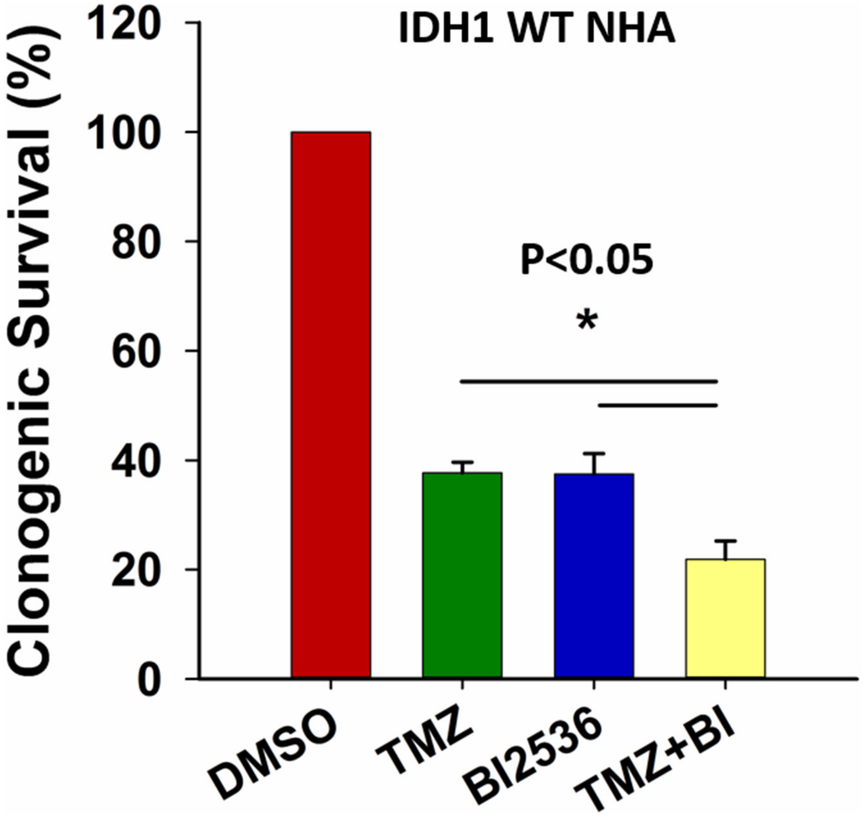

Supplementary Figure 2: Effect of TMZ and BI2536 combination treatment on IDH1 WT NHA.

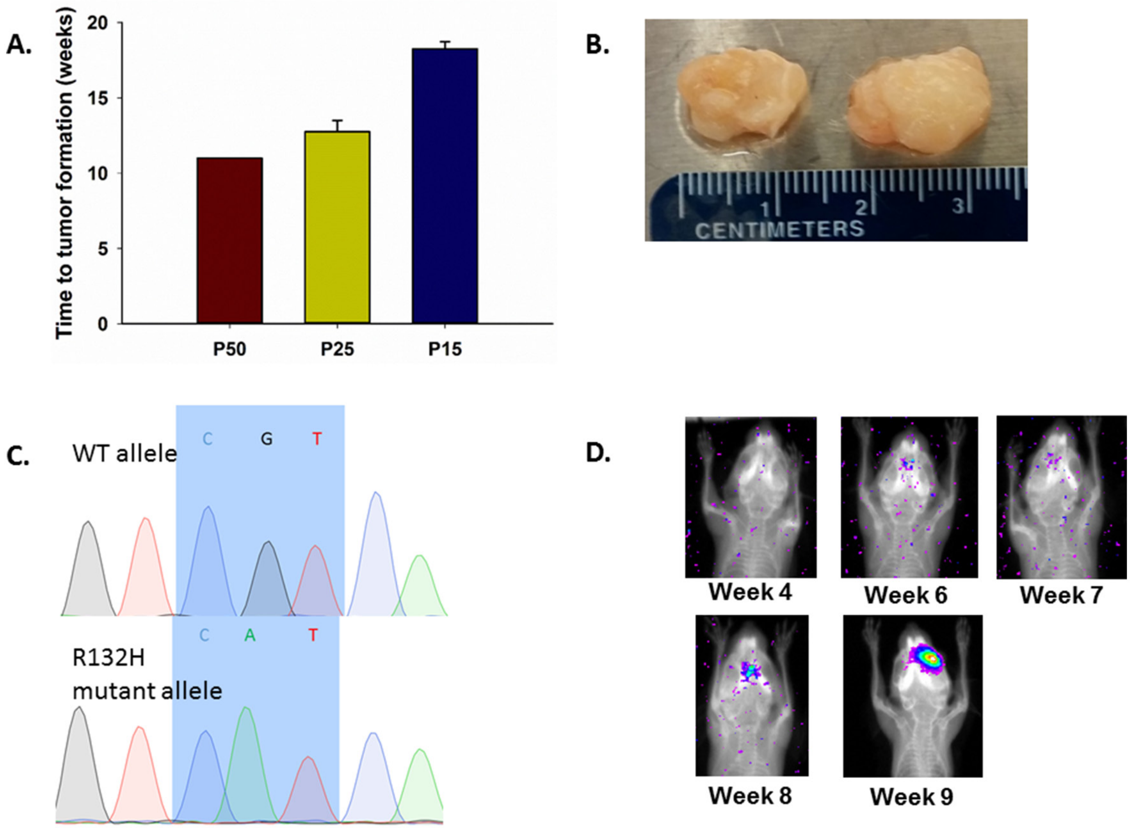

Supplementary Figure 3: *In vivo* model of IDH1-mutant glioma. A. Time to tumor formation indicates that tumor formation rate is dependent upon how long mutant IDH1 has been expressed in the cells. Time to tumor formation was determined upon formation of a 100mm^3 mass. **B.** Two representative tumors formed from P50 IDH1 mutant astrocytes 14.5 weeks after injection. **C.** Primers for the IDH1 gDNA (top) or cDNA (bottom) sequence were used to amplify endogenous and exogenous IDH1 from tumor gDNA. Targeted sequencing revealed retention of the WT (top) and R132H mutant (bottom) alleles in the subcutaneous tumors. Codon 132 is highlighted in blue. **D.** Orthotopic tumor progression monitored via luminescence. Week 4, 6, 7, and 8 scans were 20 min. Week 9 scan was 5 min.

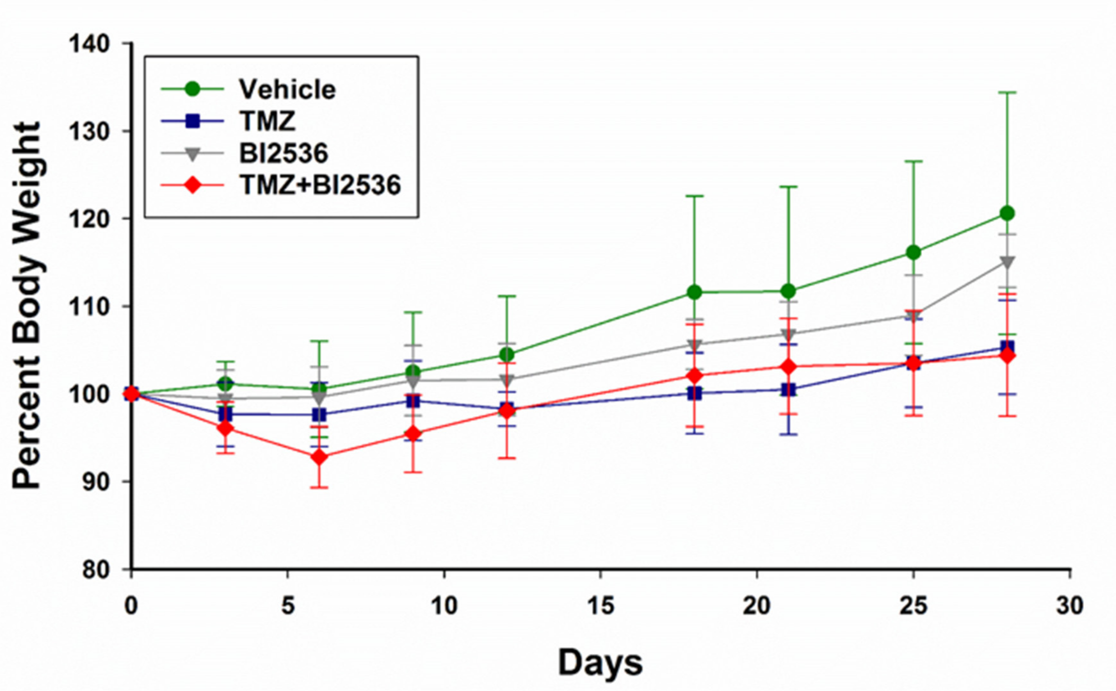

Supplementary Figure 4: TMZ+BI2536 combination therapy does not cause significant weight loss in mice. Changes in percent body weight over time of mice treated with vehicle, TMZ, BI2536, or TMZ+BI2536. Error bars show S.D.

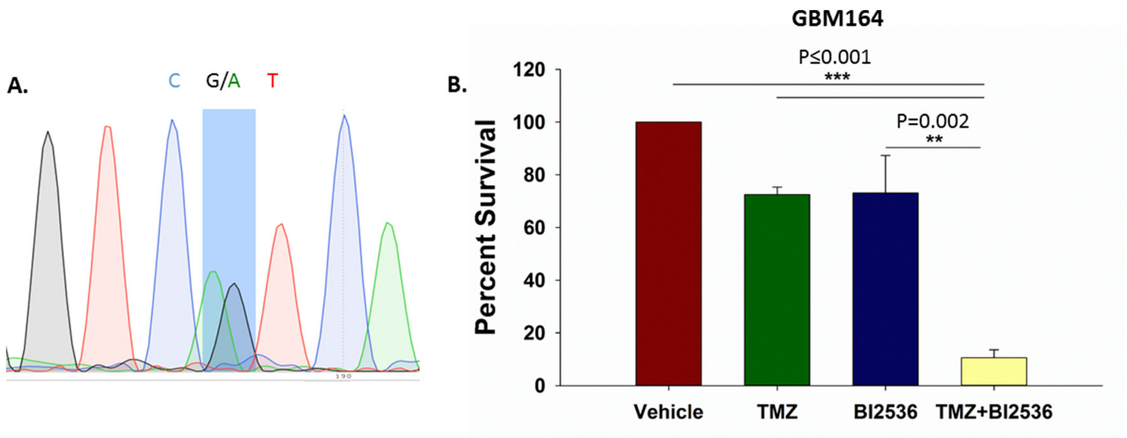

Supplementary Figure 5: BI2536 sensitizes IDH1 mutant patient-derived xenograft cells to TMZ. A. Targeted sequencing of IDH1 reveals GBM164 cells are heterozygous mutant at codon 132 (R132H mutation). The mutation site is highlighted in blue. **B.** Cell viability of GBM164 cells treated with TMZ and BI2536 singly or in combination. Error bars show SEM.
